# Development of a New Method Based on Chiral Ligand-Exchange Chromatography for the Enantioseparation of Propranolol

**Published:** 2017

**Authors:** Taher Alizadeh

**Affiliations:** *Department of Analytical Chemistry, Faculty of Chemistry, University College of Science, University of Tehran, Iran.*

**Keywords:** L-alanine, chiral separation, Propranolol, C_8_ column, Ligand exchange

## Abstract

A new chromatographic procedure was proposed for the separation of propranolol (PRN) enantiomers based upon enantioselective chiral ligand-exchange chromatography. The separation was carried out on a short C_8_ column leading to considerably short separation time. L-alanine and Cu^2+ ^were applied as chiral selector and central bivalent complexing ion, respectively. It was found that the kind of copper salt could influence the enantioseparation efficiency. The separation on the C_8_ stationary phase was more efficient than that on the C_18_ column. It was shown that the pH of mobile phase, organic modifier content of mobile phase, mole ratio of chiral ligand to bivalent ion and Cu (L-alanine) _2_ concentration in the mobile phase were important in enantioseparation efficiency. Water/methanol (70:30) mixture containing L-alanine-Cu^2+^ (7:1) was found to be the best mobile phase condition for PRN enantioseparation. All effective parameters were optimized in order to improve the separation efficiency. The optimized HPLC method was utilized for analysis of propranolol enantiomers in spiked human blood plasma samples.

## Introduction

Propranolol([1-isopropylamino-3-(1-naphthoxy)-2- propanol]) (PRN) belongs to commonly known group of β-blockers and is used to treat hypertension, sinus tachycardia, arrhythmias, coronary heart disease, and myocardial infraction where it acts preferentially upon the β-adrenergic receptors in the heart. In these compounds, (S)-enantiomers possess much greater affinity for binding to the β-adrenergic receptors rather than the (R)-antipodes (1). It has been indicated that S-(-)-propranolol is about 100 times more potent than its optical antipode (2). This indicates that there is an urgent need to develop a rapid and selective method for their enantiomeric resolution. 

High performance liquid chromatography (HPLC) separation techniques, equipped with chiral columns, have been widely utilized to chiral resolution of propranolol (3-5).

As an alternative way, indirect enantioseparation of PRN in the form of diastereomers has been carried out by RP-HPLC using a variety of chiral derivatizing reagents (6-10). 

Furthermore, thin layer chromatography was shown to be a facile technique for the separation of PRN enantiomers (11, 12). 

Other separation techniques such as micellar electro kinetic chromatography method (13) have also been utilized for the resolution of the optical isomers of propranolol. The majority of the above mentioned methods suffer from some famous disadvantages. For instance, chiral columns are expensive and usually high amounts of HPLC grade solvents are required for the elution of the desired compound from such columns. Moreover, derivatiziation reactions, required for the indirect enantioseparation is time consuming task and the control of the reaction condition such as temperature is urgently necessary to avoid racemization reactions. Therefore, further investigations for the development of more facile and simple separation techniques for propranolol enantiomeric resolution are still required.

Enantioselective ligand exchange chromatography (LEC), suggested by Davankov *et al.* (14) is a liquid chromatography technique that has provided complete, fast, cheap and reliable separation of stereoisomers of the most important classes of natural and synthetic compounds, such as amino acids, hydroxy acids, amino alcohols, and some others (15). Three kinds of the so-called LEC techniques can be identified from the literature. As the first strategy, the chiral selector is covalently attached to the stationary phase. This approach is usually called as “chiral-bonded ligand exchange” (16, 17) as the second approach, ligand exchanging columns can be easily prepared from commercially available reversed-phase columns by dynamically coating of the column with a hydrophobic chiral selector (18, 19). In the third approach, the chiral selector predominantly resides in the mobile phase. In this case, the selector complex has to be continuously introduced into the column. In such a chiral eluent, racemic mixture to be resolved, form the mixed-ligand complexes with the chiral selector. Such diastereomeric products may be differently retained through the interaction with the stationary phased packed in the column (20, 21). Major distinction of this technique is that many different chiral selectors can be easily tested with respect to the analyte to be resolved. Furthermore, small amount of ligand is usually applied as the eluent dopant, which results in enhanced rates of ligand exchange and better efficiency of the columns (22)

The mechanism of enantioselectivity in the LEC is different depending on whether the chiral ligand is linked on the stationary phase or it is added in the mobile phase as dopant (21). 

To our knowledge, there is no report on the enantioseparation of PRN by the third LEC method described above, in which a chiral dopant is continuously kept in existence in the mobile phase. In this work, we introduced L-alanine as a simply available, inexpensive and non-derivatized chiral selector for the enantioseparation of propranolol by the ligand exchange chromatography. The enantioseparation of PRN in C_8 _column was found to be better than that in C_18_, when using L-alanine as a chiral selector. It was also found that the type of copper salt, used as a source for central bivalent ion, is important in the efficiency of the enantioseparation of propranolol optically active isomers. 

## Experimental

*Reagents and instruments*

Racemic propranolol, S-(-)-propranolol and R-(+)-propranolol were purchased from Sigma-Aldrich (USA). L-alanine, Cu (CH_3_COO) _2_.H_2_O, Cu (NO_3_)_2_.3H_2_O, Cu (SO_4_)_2_.5H_2_O, Cu (Cl) _2_.2H_2_O and metanol were obtained from Merck (Germany). Chromatographic separations were carried out using a Cecil 1100 HPLC instrument, equipped with columns (125 mm Length× 4.0 mm I.D., particle size 5 μm, HiCHROM) packed with LiChrosorb RP8 and LiChrosorb RP18. Detection of eluted species was carried out by a UV detector (Cecil 1100, λ=275 nm). The mobile phase used was a mixture of methanol/water containing Cu (II) salts and L-alanine (pH=5.0). Injection volume of 15 μL was utilized in the chromatographic experiments.

*Analysis of propranolol enantiomers in human blood serum*

 In order to analyze propranolol enantiomers in plasma sample, µL amount of chiral propranolol standard solutions was injected in 5 mL plasma sample in order to adjust the propranolol concentrations in the desired levels. Then, the described plasma samples were mixed with 0.5mL of 30% ammonium hydroxide and 3 mL of 10% chloroform in n-heptane, shaken for 6 min, and centrifuged; the upper phase was evaporated to dryness under a stream of nitrogen. The residues were dissolved in 50 μL of methanol and injected to HPLC system for the analysis. 

## Result and Discussion

*Ligand exchange mechanism and enantioseparation*

In the first and second techniques of LEC (described in the introduction section of this paper) the ligand is covalently or non-covalently linked to a solid support. This allows the linked chiral selector to form diastereomeric complexes with enantiomers. These complexes may have different stabilities, leading to the racemate separation. However, the addition of chiral dopant in the mobile phase makes the mechanism of chiral resolution complicated. In such a condition it may involve a series of complexation equilibriums in the mobile and in the stationary phases as well as partition equilibriums of the different species between the two phases (21). 

In the chromatographic separation using bidentate ligand selectors, the stereoselectivity in solution is much important than the different affinity of the diastereomeric complexes to the stationary phase (23). However, in the case of tridentate ligands a different explanation for enantioseparation has been proposed (21, 24) 

Regardless of the most dominant factor involved in the separation task, the chiral ligand-exchange chromatography is based on the formation of diastereomeric ternary mixed metal complexes between the enantiomerically pure selector and the chiral isomers to be resolved. Therefore, the presence of certain functional groups, such as amino, hydroxy or carboxy groups on both selector and analyte is a prerequisite for a successful enantioseparation (21). 

In the case of PRN, two functional groups including hydroxy and amino groups are capable of creation coordination bonding with an appropriate metal ion. On the other hand, the selected chiral selector molecule (L-alanine) has also carboxylic acid and amine functional groups capable to interact with the same metal ion. Equation 1 and 2 show the proposed reactions resulting in different diastereomeric ternary mixed metal complexes.

Cu (L-alanine) _2_ + (S)-PRN [Cu (L-alanine) ((S)-PRN)] + L-alanine          (1)

Cu (L-alanine) _2_ + (R)-PRN [Cu (L-alanine) ((R)-PRN)] + L-alanine           (2)

In this study, we observed that the elution of racemic mixture of propranolol through a reversed phase column and using a mobile phase of water/methanol (70:30), doped with L-alanine and Cu (II), resulted in two separate chromatographic peaks. It was found that (R)-enantiomer was eluted before (S)-enantiomer. The peak identity was checked by injecting single (R) - and (S) - enatiomers. 

**Figure.1:**
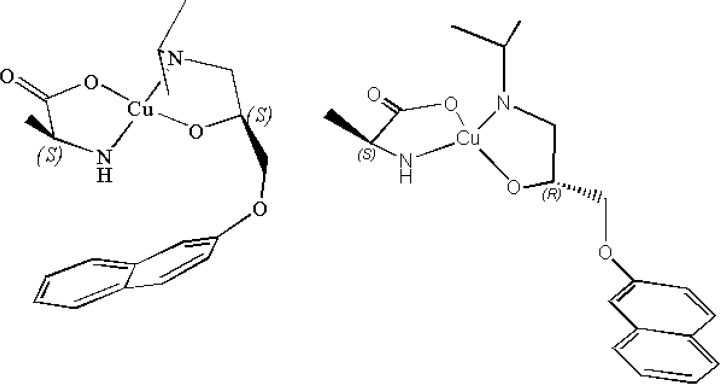
Chemical structure for the ternery diastereomeric complexes involving (R)-PRN and (S)-PRN (designed by molecular mechanics (MM2)); pseudo-homochiral complex (a), pseudo-heterochiral complex (b)

**Figure 2:**
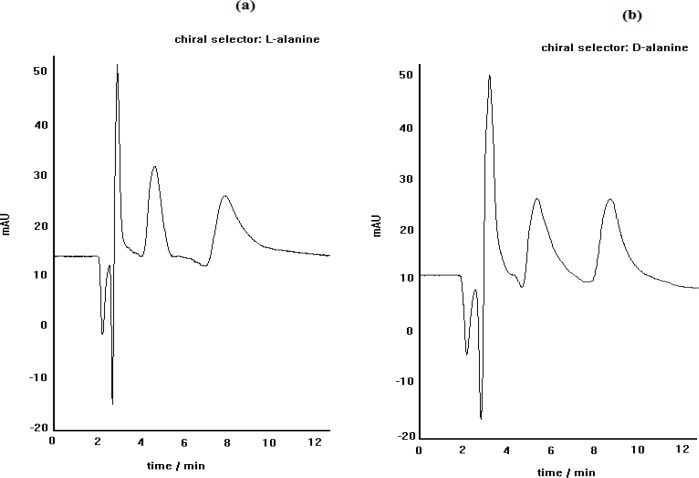
The effect of change in chiral selector kind (L-alanine to D-alanine) on the elution order of propranolol optical isomers; C_8 _column, mobile phase pH= 5, methanol/water (30:70), Flow rate= 0.4 mL.min.^-.1^

As described above, when the chiral selector is continuously doped in the mobile phase, like this procedure, the main factor involved in the recognition mechanism has been substantiated to be the affinity of the formed diastereomeric complexes to the stationary phase. The diastereomeric complexes, formed here, can show different hydrophobic interaction capability outside the complex, depending on the complex structure. The schematic representation of the suggested diastereomeric complexes are illustrated in Figure 1 The side chains of both L-alanine and (S)-PRN in the case of pseudo-homochiral complex are located in the same side in the space; whereas, the side chains of L-alanine and (R)-PRN in the case of pseudo-heterochiral complex are present in the opposite sides. This can lead to stronger hydrophobic interactions of the pseudo-homochiral complex with the apolar stationary phase (C_8_ or C_18_), resulting in longer retention time of (S)-PRN, compared to that of (R)-PRN. The described proposal was verified by replacement of chiral selectror by L-alanine with D-alanine in the mobile phase that led to the reversing of the retention order of (R)-and (S)- PRN in the column. The chromatograms depicted in Figure 2 represent the effect of change in chiral selector kind (L-alanine to D-alanine) on the elution order of propranolol optical isomers.

*The effect of copper salt type on the separation*

**Figure 3:**
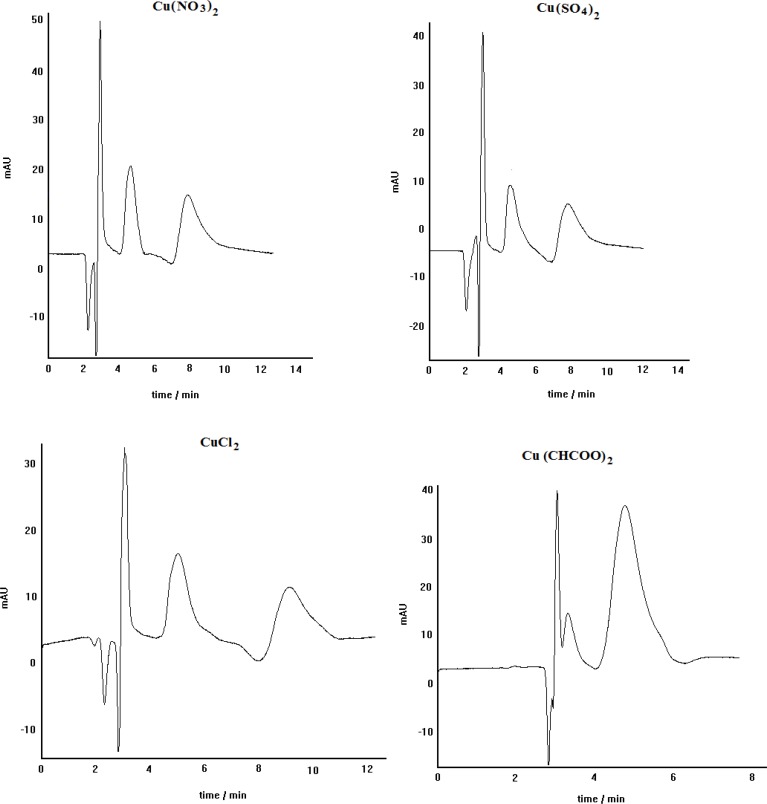
The effect of copper salt kind on the enantioseparation of propranolol by ligand-exchange chromatography: L-alanine as chiral selector, C_8 _column, mobile phase pH= 5, methanol/water (30:70), Flow rate= 0.4 mL min.^-1^

The effect of the anion of the copper salt, loaded in the mobile phase, on the enantioseparation efficiency of the LEC method has been previously reported (25-27). Furthermore, the crucial effect of copper salt kind on the enantioseparation of atenolol was described in our previous work (28). Therefore, in this work the effect of copper salt kind on the enantioseparation of propranolol by the developed method was investigated. Figure 3 represents the effect of different copper salts including copper nitrate, copper sulfate, copper chloride and copper acetate on the chiral resolution of propranolol. It can be seen that using copper nitrate, copper sulfate, and copper chloride the optically active isomers of propranolol can be resolved acceptably; although, the peaks resolution in the case of copper nitrate is relatively better than the others. However, in the case of copper acetate, no appropriate enntioseparation is identified. Each cupric anion can act as the analyte competitor in the complexation process, revealing itself more or less influent in the overall chromatographic separation. The magnitude of the stated competition has also revealed to be strictly dependent on the physico-chemical properties of each individual analyte (26). Among the examined copper salts anions, acetate anion can be considered as a powerful competitive ligand, capable of affecting the kinetic and thermodynamic of ternery diastereomeric complexes formation. We think that propranolol can not involve in the relevant diastereomeric complexes in the presence of acetate anions; because, this anion can actively participate in the complex formation, preventing thus the propranolol reaction with Cu^2+^. In the presence of the other anions, however, propranolol can participate in the relevant diastereomeric complexes formation because of weak complexing power of these anions. At such a condition, the difference in hydrophobic interactions of two formed diastereomeric complexes with nonpolar stationary phase can lead to enantiomeric separation of propranolol (28).

**Figure 4:**
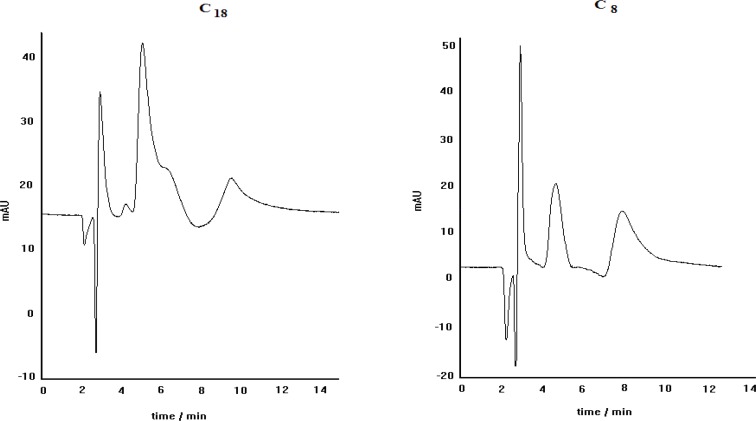
The chromatograms obtained for propranolol enantioseparation by using C_8_ (left) and C_18_ (right) columns; other separation conditions: copper nitrate as central complexing ion source, L-alanine as chiral selector, mobile phase pH=5, methanol/water (30:70) as mobile phase, Flow rate= 0.4 mL min

**Figure 5:**
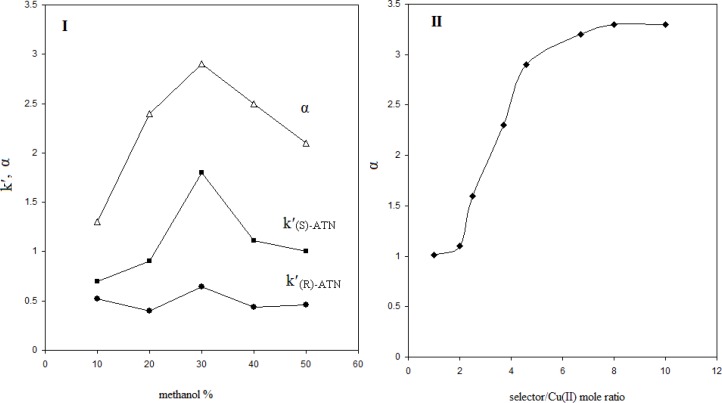
The effect of organic modifier (methanol) content of the mobile phase (I) and L-alanine/Cu^2+ ^mole ratio (II) on the PRN enantioseparation; copper nitrate as central complexing ion source, L-alanine as chiral selector, C_8_ column, mobile phase pH= 5, Flow rate= 0.4 mL min.^-1^

**Figure 6:**
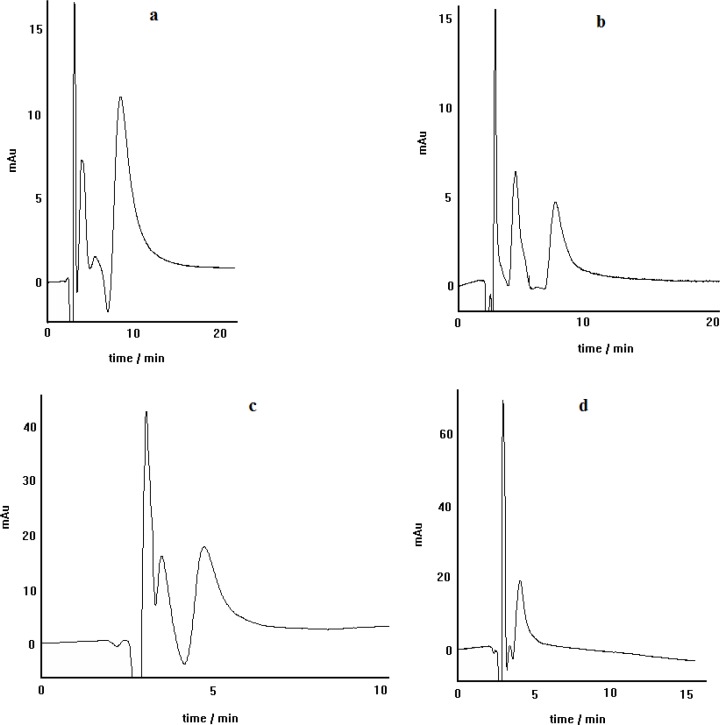
Propranolol enantioseparation by the chiral ligand exchange HPLC method using different concentrations of L-alanine including: 0.5 mmol L^-1^ (a), 1.1 mmol L^-1^ (b), 2.2 mmol L^-1^ (c) and 3.4 mmol L^-1^(d); copper nitrate as central complexing ion source, C_8_ column, mobile phase pH= 5, methanol/water(30:70) as mobile phase, Flow rate= 0.4 mL.min^-1^

**Figure 7:**
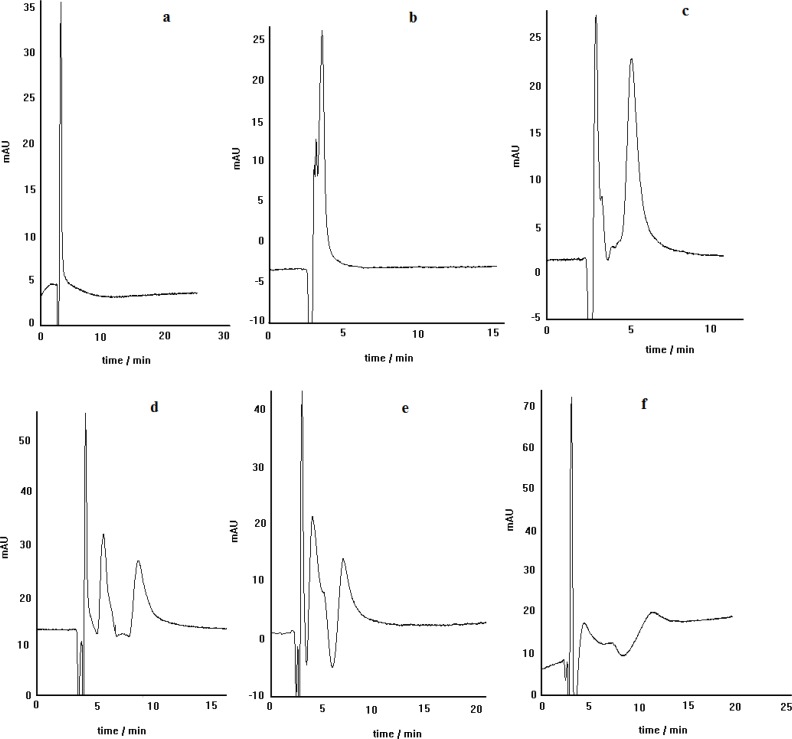
Enantioseparation of propranolol by the chiral ligand exchange HPLC method using various mobile phases pH including: 2.5 (a), 3.5 (b), 4.6 (c), 5.0 (d), 5.7 (e) and 6.7 (f); L-alanine as chiral selector, C_8_ column, Flow rate= 0.4 mL.min^-1^

**Figure 8:**
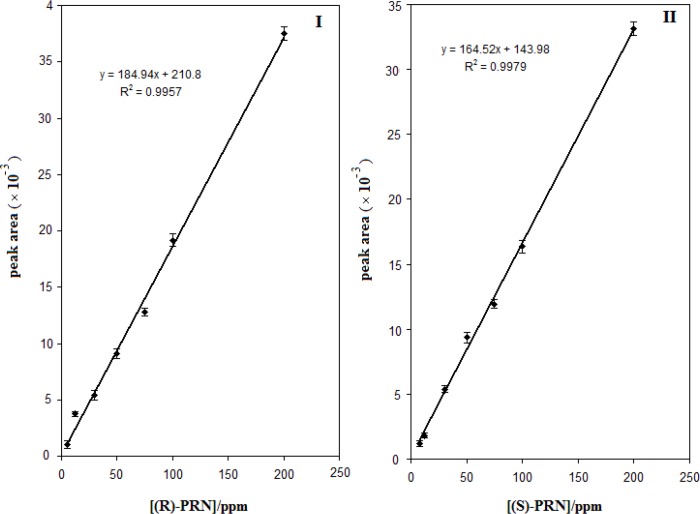
Calibration curve obtained for the (R)-PRN (I) and (S)-PRN (II) at the optimized conditions

**Table 1:** Determination of propranolol isomers in plasma samples by the developed ligand exchange chromatography method

**ample**	**add (×10** ^2^ **µg mL** ^-1^ **)**		**found (×10** ^2 ^ **µg mL** ^-1^ **)**		**recovery% ( n=4)**	
	(S)-PRN	(R)-PRN	(S)-PRN	(R)-PRN	(S)-PRN	(R)-PRN
1	12.0	12.0	12.5	12.3	104.2( ±5.4 )	102.5( ±6.5 )
2	5.5	5.5	5.2	5.1	94.5( ±6.4 )	92.7( ±5.9 )
3	15.0	5.0	14.6	4.7	97.3( ±5.9 )	94.0( ±5.7 )
4	34.0	5.0	35.5	5.4	105.0( ±6.1 )	104.4( ±5.1 )

*The effect of column on the separation*

The effect of stationary phase kind on the separation efficiency was examined by carrying out of propranolol enantioseparation via two different columns, packed with C_8_ and C_18_ stationary phases. The obtained results are demonstrated in Figure. 4. As can be seen, the separation of PRN enantiomers by ligand-exchange chromatography technique in C_8 is_ better than that in C_18_, regarding the peak resolution efficiency. It is clear that both peaks in C_18_ column are broadened and the separation of two enantiomers is not satisfactory. These results indicate that the stationary phase type is a prominent factor in enatioseparation; although, both stationary phases are inherently achiral and provide the same interaction nature, when encountering the species to be eluted. As a probable proof for this observation, we think that in the case of C_18_ phase, non-selective retention of the non-complexed PRN makes a noticeable contribution in the observed inappropriate separation process.

*The effect of organic modifier and selector/Cu (II) mole ratio*

It was found that the presence of organic solvent was crucially necessary for the enantioseparation of PRN; since, the separated peaks were highly broadened in the absence of organic solvent in the mobile phase. No appropriate resolutions were obtained by using acetonitrile and tetrahydrofurane. However, the addition of methanol, as an organic modifier, to the mobile phase resulted in reliable enantioseparation. Furthermore, the amount of methanol in the mobile phase was also found to be important, regarding the chiral separation efficiency. Figure 5 (I) shows the effect of methanol percent in the mobile phase on the separation parameters including K′_R_, K′_S_ and α. It can be seen that K′_S_ is influenced more intensively by the methanol amount changing, compared to K′_R_. This is reasonable; since, the interaction of (S)-PRN involved ternary complex with the stationary phase is higher than that formed with (R)-PRN. This parameter increases firstly, as the methanol percent increases; but, after 30%, further increase in methanol amount gives rise to decrease in K′_S_. Such a behavior can be observed in the case of K′_R_; however, the dependence of the later parameter on the methanol amount is not considerable. The described figure indicates that the best separation can be obtained in methanol percent of 30%, regarding the α value which is maximum when the percent of methanol reaches to 30% in the mobile phase composition. 

Figure 5 (II) shows that as the mole ratio of (L-alanine/Cu^2+^) increases in the mobile phase, the selectivity coefficient sharply enhances till mole ratio of about 7 and after, the enantioselectivity changing as a function of (L-alanine/Cu^2+^) mole ratio is not noticeable. 

It was observed that other than the described mole ratio the amount of chiral selector, loaded in the mobile phase, was critically important in the efficiency of the chiral separation of propranolol. Figure 6. Shows the effect of the concentration of L-alanine in the mobile phase on the enantioseparation performance. It must be noted that in this experiment the mole ratio of (L-alanine/Cu^2+^) was fixed at the value of 7. It can be seen that this factor has an important role in the efficiency of the enantioseparation. It is evident that one need to an optimum amount of chiral selector for successful enantioseparation, namely, above and also below the optimum amount, the enantioseparation efficiency of the developed method is lost. The results depicted in Figure 6, suggest that using 1.1 mm of L-alanine in the mobile phase can lead to a satisfactory propranolol enantioseparation.

*The effect of mobile phase pH*

The dependence of propranolol enantioselectivity on the mobile phase pH was investigated by changing mobile phase pH in the range of 2.5-6.7. Figure 7 show the chromatograms obtained by the developed chiral separation method at different mobile phase pH values. It is clear that the mobile phase pH has crucial effect on the enantiomers resolution. As can be observed, when the pH of mobile phase is adjusted to 2.5, 3.5 or even 4.6 no resolution is observed for PRN enantiomers. This may be because of suppression of the formation diastereomeric complexes at lower pH values. The enantioseparation of propranolol is achieved at pH of about 5. Further than pH of 5, the enantioseparation is still retained; but, it is not as proper as that at pH of 5. Therefore, in all separation experiments, executed in this work, the mobile phase pH was adjusted to about 5. 

*Validation of the method*

In order to apply the developed enantioseparation method for quantitative analysis of propranolol enantiomers the calibration curves were constructed using the areas of the chromatographic peaks of both enantiomers, calculated at increasing concentrations. The results obtained are illustrated in Figure.8 .Based on the depicted curves, the (S)-PRN and (R)-PRN can be determined at linear concentration ranges of 5-200 and 8-200 µg mL^-1^, respectively. The limits of detections for (S)-PRN and (R)-PRN were found to be 2.0 and 1.1 µg mL^-1^, respectively (signal-to-noise ratio of 3). The intra- and interday accuracy and precision of the assay, assessed as relative standard deviation (RSD), were determined by assaying the samples of PRN enantiomers at three different concentrations in four replicates in the same day and consecutive days. The results showed that the intraday relative standard deviations and interday relative standard deviations of the proposed method were 4.3% and 5.4% for (S)-PRN, and 3.3% and 5.4% for (R)-PRN, respectively. 

The developed HPLC method was applied for the analysis of propranolol enantiomers in the spiked human blood plasma samples. The obtained results are summarized in table 1. It must be noted that, before spiking of propranolol, the presence of any propranolol in the tested sample was checked by using the developed HPLC method and the absence of propranolol in aimed plasma samples was confirmed. It can be seen that the method leads to satisfactory results in propranolol enantiomers measurement in plasma samples, regarding recoveries and determination precision in all tested cases. 

## Conclusion

A new HPLC method based upon ligand-exchange chromatography on a C_8_ column was developed for the effective enantioseparation of PRN. Using a short column led to relatively short separation time. L-alanine was shown to be an effective chiral selector in the PRN enantioseparation. It was demonstrated that the copper salt kind, used as a central complexing ion source, had distinct effect on the enantioseparation efficiency of PRN. No effective separation was observed using acetate copper; however, other copper salts like copper nitrate, copper sulfate and copper chloride led to propranolol enantioseparation. Moreover, the separation on C_8_ column was found to be more effective than that in C_18_ column, despite the similarity between interaction nature of C_8_ and C_18_ columns. The concentration of L-alanine, L-alanine/Cu (II) mole ratio and the organic modifier percent were found to be important in the separation efficiency. Mobile phase pH had also crucial effect on the separation efficiency. The optimized method was utilized successfully for the analysis of propranolol enantiomers in plasma samples.
